# Loss of protein tyrosine phosphatase receptor delta PTPRD increases the number of cortical neurons, impairs synaptic function and induces autistic-like behaviors in adult mice

**DOI:** 10.1186/s40659-024-00522-0

**Published:** 2024-06-18

**Authors:** Bastián I. Cortés, Rodrigo C. Meza, Carlos Ancatén-González, Nicolás M. Ardiles, María-Ignacia Aránguiz, Hideaki Tomita, David R. Kaplan, Francisca Cornejo, Alexia Nunez-Parra, Pablo R. Moya, Andrés E. Chávez, Gonzalo I. Cancino

**Affiliations:** 1 Laboratorio de Neurobiología, Facultad de Ciencias Biológicas, Pontificia Universidad Católica de Chile, Santiago, 8331150 Chile; 2 Centro Interdisciplinario de Neurociencia de Valparaíso (CINV), Facultad de Ciencias, Universidad de Valparaíso, Valparaíso, 2340000 Chile; 3 Programa de Doctorado en Ciencias mención Neurociencias, Facultad de Ciencias, Universidad de Valparaíso, Valparaíso, 2340000 Chile; 4 Program in Neuroscience and Mental Health, The Hospital for Sick Children, Toronto, M5G 1X8 Canada; 5 Department of Molecular Genetics, University of Toronto, Toronto, M5S 1X8 Canada; 6 Center for Integrative Biology, Facultad de Ciencias, Universidad Mayor, Santiago, 8580745 Chile; 7 Cell Physiology Laboratory, Biology Department, Faculty of Science, Universidad de Chile, Santiago, 7800003 Chile; 8 Centro de Estudios Traslacionales en Estrés y Salud Mental (C-ESTRES), Universidad de Valparaíso, Valparaíso, 2340000 Chile; 9 Instituto de Fisiología, Facultad de Ciencias, Universidad de Valparaíso, Valparaíso, 2340000 Chile; 10 Instituto de Neurociencias, Facultad de Ciencias, Universidad de Valparaíso, Valparaíso, 2340000 Chile; 11 Ludna Biotech Co., Ltd, Suita, Osaka, 565-0871 Japan

**Keywords:** Protein tyrosine phosphatase receptor delta, Medial prefrontal cortex, Synaptic transmission, Anxiety, Learning, Memory, ASD

## Abstract

**Background:**

The brain cortex is responsible for many higher-level cognitive functions. Disruptions during cortical development have long-lasting consequences on brain function and are associated with the etiology of brain disorders. We previously found that the protein tyrosine phosphatase receptor delta Ptprd, which is genetically associated with several human neurodevelopmental disorders, is essential to cortical brain development. Loss of *Ptprd* expression induced an aberrant increase of excitatory neurons in embryonic and neonatal mice by hyper-activating the pro-neurogenic receptors TrkB and PDGFRβ in neural precursor cells. However, whether these alterations have long-lasting consequences in adulthood remains unknown.

**Results:**

Here, we found that in *Ptprd+/-* or *Ptprd-/-* mice, the developmental increase of excitatory neurons persists through adulthood, affecting excitatory synaptic function in the medial prefrontal cortex. Likewise, heterozygosity or homozygosity for *Ptprd* also induced an increase of inhibitory cortical GABAergic neurons and impaired inhibitory synaptic transmission. Lastly, *Ptprd+/-* or *Ptprd-/-* mice displayed autistic-like behaviors and no learning and memory impairments or anxiety.

**Conclusions:**

These results indicate that loss of *Ptprd* has long-lasting effects on cortical neuron number and synaptic function that may aberrantly impact ASD-like behaviors.

## Introduction

The cerebral cortex is responsible for processing complex sensory and cognitive behaviors, requiring an extraordinary control over the generation of neurons and their connections during the development of the nervous system [1]. Typically, cortical circuits are assembled from different glutamatergic and GABAergic neuron lineages, each with unique morphological and physiological characteristics [2, 3]. Indeed, disruption in the generation of neurons or their connectivity has long-lasting consequences affecting behavior that can result in brain disorders. In this regard, it has been proposed that the number, diversity, and function of glutamatergic and GABAergic neurons are essential to maintain the balance between different cerebral circuits and to avoid the onset of multiple neuropsychiatric conditions [4]. Perturbation of cellular processes such as neural precursor cell proliferation, neurogenesis, and neuronal migration during brain development may, in part, cause structural brain alterations that lead to the onset of brain disorders [5]. Gain or loss of expression or activity of genes involved in these processes are associated with the onset of several brain disorders, including autism spectrum disorder (ASD), schizophrenia, bipolar disorder, attention-deficit/hyperactivity disorder (ADHD), and intellectual disability [5–10]. We reported that alterations in the expression of one of these genes, protein tyrosine phosphatase receptor delta (PTPRD), impairs cerebral cortex development in mice [11, 12].

Ptprd is a receptor protein tyrosine phosphatase type II family member. Structurally, it contains extracellular immunoglobulin and fibronectin III domains and two cytoplasmic tyrosine phosphatase domains [13–15]. We reported that *Ptprd* hetero- or homozygosity during cortical development triggered dysregulation of TrkB and PDGFRβ tyrosine kinase activity in embryonic and neonatal cortical neural precursor cells. Ptprd acted as a TrkB and PDGFRβ phosphatase, fine tuning kinase activity and preventing their hyperactivation. Loss of *Ptprd* resulted in aberrantly increased proliferation of neural precursor cells and concomitant mispositioning of their progeny, embryonic and newborn excitatory neurons [11, 12]. Ptprd regulation of receptor tyrosine kinases like TrkB thus ensures the generation of appropriate numbers of neural precursor cells and neurons postnatally. Ptprd also performs a critical function at synapses, regulating cell adhesion important for synaptic formation and differentiation [15]. Given the role of Ptprd in assuring appropriate numbers of postnatal cortical neuron subtypes and its role at synapses, we asked if alterations of cortical neuron numbers observed embryonic and postnatally persist into adulthood, and if they perturb cortical synaptic function and ultimately, behavior.

Studies of how *Ptprd* deficiency alters excitatory and inhibitory synaptic transmission have yielded contradictory results [16–19]. For example, in hippocampal neuron cultures, Ptprd is involved in inhibitory synaptic formation and transmission but not in the development of excitatory synaptic transmission [16]. More recently, the loss of *Ptprd* expression differentially suppressed excitatory synaptic transmission in the stratum lacunosum moleculare in the hippocampus but not in the stratum radiatum [17]. Moreover, deletion of *Ptprd* in cultured neurons did not alter the development or number of synapses [18] and did not interfere with excitatory or inhibitory synaptic development and transmission [19]. It is important to note that these findings focused on hippocampal neurons. Whether the absence of PTPRD alters synaptic transmission in cortical neurons has not yet been addressed.

Here we address these issues by assessing in adult *Ptprd-/-* mice the number of glutamatergic and GABAergic neurons in the brain cortex, recording both excitatory and inhibitory synaptic function and analyzing animal behavior. Our results indicate that ablation of *Ptprd* results in an increase in both glutamatergic and GABAergic neurons in adult cortex leading to changes in excitatory and inhibitory synaptic transmission. Moreover, *Ptprd+/-* or *Ptprd-/-* mice show deficits in sociability and social novelty, and repetitive behavior characteristic of ASD-like behaviors when compared to *Ptprd+/+* mice. These findings indicate that loss of one or both alleles of *Ptprd* during the formation of the cerebral cortex leads to profound structural changes that affect the assembly and functioning of glutamatergic and GABAergic circuits and synaptic transmission, likely contributing to ASD-like behaviors in adulthood.

## Methods

### Animals

The C57BL/6 N-A < tm1Brd > *Ptprd* < tm2a(KOMP)Wtsi>/WtsiOrl mice strain was purchased from Wellcome Trust Sanger Institute and maintained as heterozygous. The cross between heterozygous mice allowed the *Ptprd+/+*, *Ptprd+/-*, and *Ptprd-/-* mice to be obtained. The following primers were used to identify the three genotypes mentioned above: Ptprd_111547_F: 5’-TCACCTCGCTGTTCTTCCTG-3’; Ptprd_111547_R:5’-CTTCTCAGTGCCCAACCCTC-3’; CAS_R1_Term: 5’-TCGTGGTATCGT TATGCGCC-3’. In addition, for sociability and social novelty tests, BALB/c wild-type mice were used as “old mouse” and “new mouse”. All mice had free access to rodent chow and water in a 12-hour dark-light cycle room with temperature controlled at 20–22 °C.

### Perfusion of mice and brain extraction

*Ptprd+/+*, *Ptprd+/-*, and *Ptprd-/-* mice were perfused transcardially at 3 months old. The mice were first placed in an isoflurane chamber to be lightly anesthetized. Once the animal was numb, the heart was exposed, and a saline solution (0.9% NaCl) was injected gradually through the right ventricle. Once the blood flow stopped, the brains were extracted and fixed in a 4% PFA solution overnight at 4 °C. The next day, the brains were dehydrated in 20%, 25%, and 30% sucrose (Merk, 107,651,100) solutions at 4 °C for 24 h each incubation. Subsequently, brains were embedded in the optimal cutting temperature compound (OCT; Sakura, 4583) and stored at -80 °C until cut. Next, 18 μm coronal slices were obtained by cryostat (Leica, CM1850) at a temperature of -20 °C and stored at -80 °C until use.

### Immunofluorescence and quantification

For morphometric analysis, immunostaining of tissue sections was performed as described [11, 12, 20]. Briefly, brain sections were washed with 1X TBS for immunostaining. Then were permeabilized with TBS − 0.3% Triton X-100 for 30 min. The tissues were then incubated in TBS with 5% BSA 0.3% Triton X-100 for 1 h as a blocking solution. Brain slices were incubated overnight with primary antibodies in a blocking solution at 4 °C. The next day, the sections were washed with TBS and then incubated with secondary antibodies in a blocking solution for 1 h at room temperature. After TBS washes, sections were counterstained with Hoechst 33,258 for 10 min and mounted. To evaluate the number of GABAergic neurons in the somatosensory cortex and medial prefrontal cortex (mPFC), rabbit anti-parvalbumin antibody (1:100; Swant, PV 25) and mouse anti-somatostatin antibody (1:500; Santa Cruz, sc-55,565) were used. To evaluate the number of glutamatergic neurons in the somatosensory cortex and mPFC, rabbit anti-Tbr1 antibody (1:400; Abcam, ab31940) and rabbit anti-Satb2 antibody (1:500; Abcam, ab92446) were used. To quantify the specific neuronal types, the somatosensory cortex and mPFC boundaries were established using the mouse brain Atlas [21] and Adobe Photoshop CC 2015. Then, the images were quantified using the Cell Counter tool of the Fiji program.

### Prefrontal cortex slice preparation

Acute coronal brain slices (300 μm thick) were obtained as previously described [22]. Briefly, the brain was removed and slices containing the mPFC were cut using a DTK-1000 Microslicer (Ted Pella, Inc.) in an ice-cold high-choline solution (110 mM Choline-Cl, 2.5 mM KCl, 1.25 mM NaH_2_PO_4_, 7 mM MgCl_2_, 25 mM NaHCO_3_, 15 mM glucose, 0.5 mM CaCl_2_, 11.6 mM ascorbate, 3.1 mM pyruvate (290–305 mmol/Kg)). Thirty minutes post-sectioning, coronal slices were gradually switched to artificial cerebral spinal fluid (aCSF) solution (124 mM NaCl, 2.69 mM KCl, 1.25 mM KH_2_PO_4_, 1.3 mM MgSO_4_, 26 mM NaHCO_3_, 10 mM glucose, 2.5 mM CaCl_2_, pH 7.4 (300–305 mmol/Kg)). All solutions were equilibrated with 95% O_2_ and 5% CO_2_ (pH 7.4), and slices were kept at room temperature for at least 30 min before recording.

### Electrophysiology

All experiments, unless otherwise stated, were performed at 28 ± 1 °C in a submersion-type recording chamber perfused at ~ 2 mL/min with aCSF supplemented with either the GABAA receptor antagonist picrotoxin (PTX; 50 µM) or the AMPA/Kainate and NMDA receptor antagonists CNQX (25 µM) and D-APV (50 µM) to block fast inhibitory and excitatory transmission, respectively. Coronal slices were visualized using infrared differential interference contrast on a Nikon Eclipse FN1 microscope, and layer 2/3 pyramidal neurons from prelimbic (PrL) and infralimbic (IL) subdivisions of mPFC were morphologically identified. Whole-cell voltage-clamp recordings using a Multiclamp 700B amplifier (Molecular Devices, Sunnyvale, CA, USA) were made from 2/3 pyramidal neuron somas located ~ 200 mm from pia and voltage-clamped at -60 or 0 mV (unless otherwise stated) using patch-type pipette electrodes (~ 3.0–4.5 MΩ) containing 131 mM Cs-gluconate, 8 mM NaCl, 1 mM CaCl_2_, 10 mM EGTA, 10 mM glucose, 10 mM HEPES, 5 mM MgATP, and 0.4 mM Na_3_GTP; pH 7.2–7.4 (285 mmol/kg). To assess cell stability, series, and input resistances were monitored with test pulses (-4mV, 80 ms) throughout all experiments. Cells with > 20% change in series resistance were excluded from the analysis.

To elicit excitatory and inhibitory postsynaptic currents (EPSCs and IPSCs, respectively), a bipolar-stimulating patch pipette filled with aCSF was placed in layer 2/3 around 100–150 μm from the recording neuron and voltage pulses (1–15 V, 100–200 ms pulse width) were delivered through a stimulus isolator (DS2A-Mk.II, Digitimer Ltd). Typically, stimulation was adjusted to obtain comparable magnitude synaptic response across experiments (e.g., 100–150 pA EPSC and IPSC). Two different protocols were used to evaluate short-term synaptic plasticity: First, two pulses at different interstimulus intervals (10, 30, 70, 100, and 300 ms) were used to calculate the paired-pulse ratio (PPR) that was defined as the ratio of the amplitude of the second response to the amplitude of the first response. Second, synaptic depression was evaluated using a burst of 25 (for EPSC) and 20 (for IPSC) stimuli at 14 and 10 Hz, respectively, and delivered every 60 s; 5 burst-evoked responses were averaged for each experiment. Miniature EPSCs (mEPSCs) were recorded at 32 ± 1 °C from neurons voltage-clamped at -60 mV in the continuous presence of tetrodotoxin (TTX, 500 nM). In contrast, isolated miniature IPSCs were recorded at 0 mV in the continuous presence of TTX, CNQX (25 µM), and D-APV (50 µM). mEPSCs and mIPSC were identified using a minimal threshold amplitude (≥ 5 pA) and analyzed using the mini-analysis software Synaptosoft (Synaptosoft). Spontaneous excitatory and inhibitory currents (sEPSCs/sIPSCs) were recorded without TTX, whereas AMPAR/NMDAR ratios were analyzed by recording AMPAR-mediated EPSCs at -60 mV. In contrast, NMDAR-mediated EPSCs were recorded at + 40mV in the continuous presence of CNQX (25 µM) to isolate NMDA-mediated EPSC. All drugs were obtained from Sigma and Tocris, prepared in stock solutions in milli-Q water or DMSO, and added to the ACSF as needed. Total DMSO in the aCSF was maintained at > 0.1%. Synaptic EPSCs and IPSCs were elicited at 10-sec intervals (0.16 Hz), filtered at 2.2 kHz, and acquired at 10 kHz using custom-made software written in Igor Pro 4.09 A (WaveMetrics).

### Morris water maze test

Morris water maze navigation task was performed to evaluate spatial learning and memory as previously described [23]. Briefly, in the learning phase, the animals were trained to locate a hidden 9-cm-diameter white platform (1 cm below the surface of the water) for 4 consecutive days following 4 external cues, 4 times per day per animal in a 1.2-m-diameter circular pool filled with 23 ± 2 °C painted white water. Each trail ended when the animals found the platform and remained there for 10 s. If the animal did not find the platform at the end of 60 s, they were gently guided there for the remaining 10 s. Later in each trial, the animals were gently removed from the maze, dried with a tissue paper towel, and put in a similar housing cage with additional tissue paper towels to continue drying. On the fifth day, in the memory phase, the test was performed by removing the platform and allowing the free swimming for 60 s. The behavior was monitored during all tasks using an automatic tracking system (ANY-maze video tracking software, Stoelting Co, Wood Dale, IL, USA). The learning phase measured latency and path length to the platform, while the memory test measured the time spent in each quadrant and the target quadrant and the traveled distance within the target quadrant.

### Y-Maze test

Memory was also evaluated with the Y-maze as previously described [24]. Briefly, Y-Maze tests were performed using a maze with 3 arms of 8 cm width x 35 cm long and walls of 20 cm height, connected at a 120° angle, shaping a Y and assigned as arms A, B, and C. Each animal was placed in the arm (A), looking to the center of the maze, and tracked for 8 min by the ANY-maze software. Four clues were put on the maze walls to guide the animals. Alternation was defined as a visit to 3 different arms (ABC, ACB, BCA, BAC, CAB, CBA), and % spontaneous alternation was determined by the following formula:

% Spontaneous alternation = (Total alternations / (Total arm entries-2) x 100.

### Open field test

Anxiety was evaluated with the open field test as described previously [25]. Briefly, mice were exposed to a free exploration in an open field chamber of 40 cm (l) x 40 cm (w) x 40 cm (h). This arena is divided into two areas by a line: the peripheral and central regions. The time in each chamber area was recorded for 10 min by ANY-maze software.

### Elevated plus maze test

Anxiety was also evaluated with the evaluated plus maze as described previously [26]. Briefly, the test was performed using a maze with 4 arms of 7.5 cm width x 35 cm long, shaping a cross with a 7.5 × 7.5 cm center and 50 cm from the floor. 2 arms (closed arms) had walls of 20 cm height, and the others (open arms) were uncovered. The animals were placed in the center of the maze and tracked for 5 min by ANY-maze software.

### Self-grooming test

Repetitive behavior was measured by the self-grooming test as previously described [25, 27]. Briefly, mice were recorded for 10 min through a 20 × 20 cm transparent box to allow visualization of the animal. The cumulative time spent by the animals performing the self**-**grooming process was quantified.

### Marble burying test

Repetitive behavior was also evaluated by the marble burying test as previously described [22, 25]. Briefly, the animals were exposed to a free exploration arena of 23 × 33 cm in the presence of 20 proportionally distributed black marbles. The marbles were placed in a 5 cm thick layer of dry sawdust to allow the animal to perform the digging behavior. After 30 min, the number of buried marbles was quantified. A marble was considered buried if 2/3 were covered.

### Three-chamber test

The three-chamber social novelty and sociability test was used to evaluate deficits in social behavior as previously described [25, 27, 28]. First, the animals were subjected to 10 min of habituation in the three chambers. Then, to assess the sociability of the animals, mice were placed in a context in which they chose to interact with either a caged mouse (old mouse) or an inanimate object, each in a different chamber for 10 min. Subsequently, to assess the social novelty, the inanimate object was replaced by a second unfamiliar mouse (new mouse), and the experimental mouse was placed in a context in which it had to choose between interacting with a familiar or an unfamiliar mouse for 10 min. ANY-maze software recorded the time the experimental animals spent with each option.

### Statistical analyses

Statistical analyses were performed using the one-way ANOVA test with unpaired data to compare data between *Ptprd*+/+, *Ptprd*+/-, and *Ptprd*-/- genotypes. Unless otherwise indicated, all electrophysiological results were statistically analyzed using paired and unpaired two-tailed Student’s t-test and One- or Two-way ANOVA at the *p* < 0.05 significance level in OriginPro 7.0 and 8.6 (OriginLab). In the figures illustrated, traces are averages of 25–30 responses. In the case of the statistical analysis used to evaluate the sociability and social novelty test, the 2-way ANOVA test was used. Corrections for multiple comparisons were performed using Tukey’s post hoc test. All statistical tests were performed with Prism 6 software. In all cases, the bars indicate the standard error of the mean.

## Results

### *Ptprd*+/- or *Ptprd*-/-mice display an increase in glutamatergic neurons and impaired synaptic function in the adult cortex

**Fig. 1:**
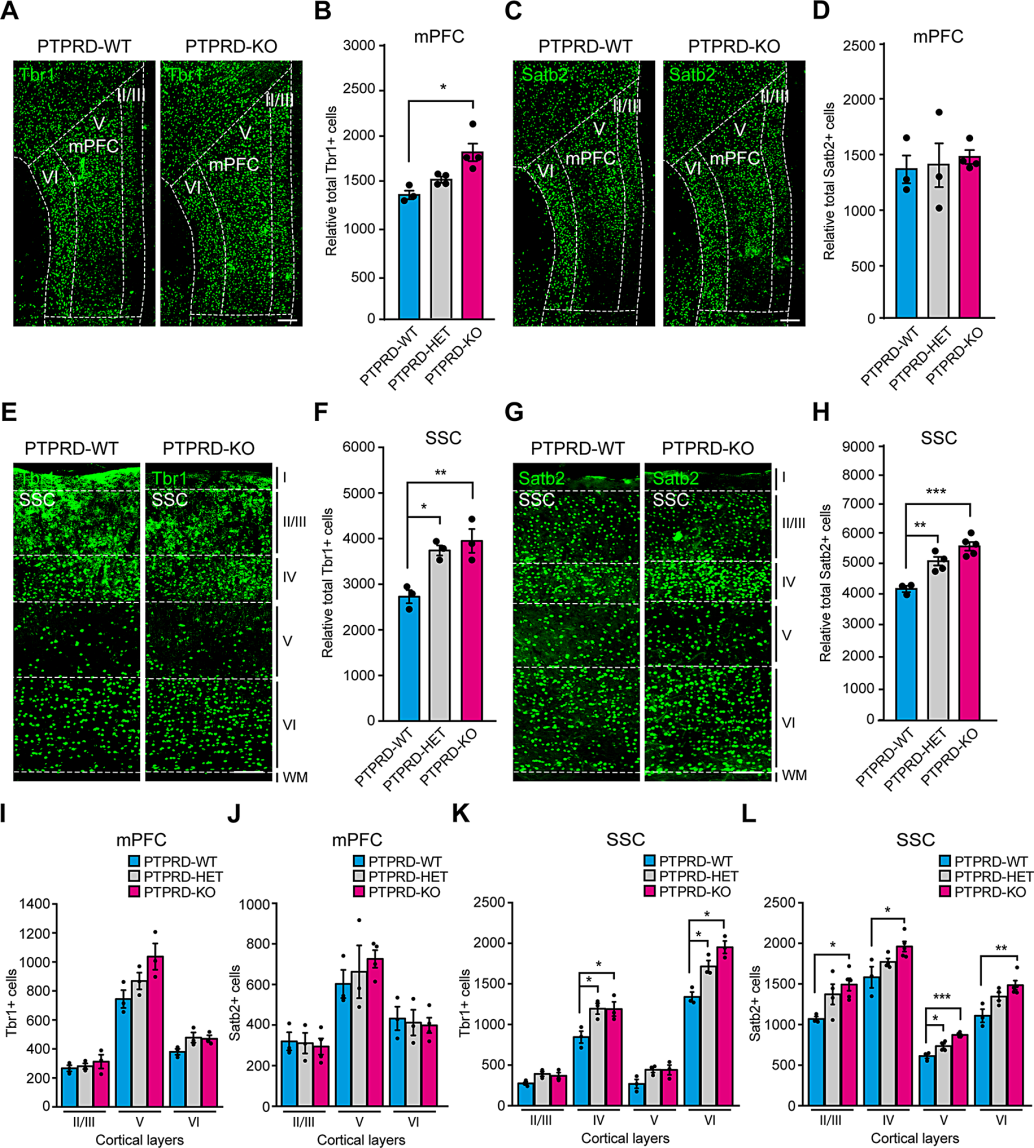
Adult *Ptprd+/-* or *Ptprd-/- *mice show an increase of glutamatergic neurons in the mPFC and somatosensory cortex. (**A-H**) Coronal sections of mPFC (**A, C**) or somatosensory cortex (**E, G**) of *Ptprd*+/+ (PTPRD-WT) and *Ptprd*-/- (PTPRD-KO) 3-month-old animals were immunostained against Tbr1 (**A, E**) or Satb2 (**C, G**). Relative total Tbr1-positive cells in the mPFC **(**for **B**, PTPRD-WT, *n* = 3; PTPRD-HET, *n* = 4; PTPRD-KO, *n* = 4**)** or somatosensory cortex **(**for **F**, PTPRD-WT, *n* = 3; PTPRD-HET, *n* = 5; PTPRD-KO, *n* = 5**)** of *Ptprd*+/+, *Ptprd*+/- (PTPRD-HET), and *Ptprd*-/- 3-month-old mice were quantified. Relative total Satb2-positive cells in the mPFC **(**for **D**, PTPRD-WT, *n* = 3; PTPRD-HET, *n* = 3; PTPRD-KO, *n* = 4**)** or somatosensory cortex **(**for **H**, PTPRD-WT, *n* = 3; PTPRD-HET, *n* = 4; PTPRD-KO, *n* = 5**)** of *Ptprd*+/+, *Ptprd*+/-, and *Ptprd*-/- 3-month-old mice were quantified. In (**A**) and (**C**), the dotted line shows mPFC and specific cortical layers. **(I, J)** Tbr1 (**I**) or Satb2 (**J**) positive neurons were quantified per cortical layers II/III, V and VI in the mPFC. **(K, L)** Tbr1 (**K**) or Satb2 (**L**) positive neurons were quantified per cortical layers II/III, IV, V and VI in the somatosensory cortex. mPFC = medial prefrontal cortex. SSC = somatosensory cortex. WM = white matter. *p < 0.05; **p < 0.01; ***p < 0.001. In all the images, scale bars represent 200 μm. In all graphs, the error bars denote SEMs

We previously reported that *Ptprd+/-* and *Ptprd-/-* embryos have more excitatory cortical neurons [11]. To determine whether this phenotype persisted until adulthood, we performed immunostaining for Tbr1 (Fig. 1A) and Satb2 (Fig. 1C), markers of deep- and upper-layer excitatory cortical neurons, respectively, in coronal sections of the mPFC, a brain area involved in different cognitive processes, from 3-month-old *Ptprd+/+*, *Ptprd+/-*, and *Ptprd-/-* mice. While heterozygous mice showed no differences as compared to control littermates, *Ptprd-/-* mice had 20% more Tbr1-positive neurons than control and *Ptprd+/+* littermates in the mPFC (Fig. 1B). However, we did not observe any changes in the number of Satb2-positive neurons (Fig. 1D), suggesting that only early born neurons are affected at the mPFC. To complement this result, we analyzed the somatosensory cortex, another cortical region commonly disrupted in brain disorders associated with *PTPRD* mutations. We found that *Ptprd-/-* and *Ptprd+/-* mice had more Tbr1-(Fig. 1E, F) and Satb2-positive excitatory neurons (Fig. 1G, H).

The brain cortex has 6 layers which are connected to different brain circuits. To determine if this increase in excitatory neurons is cortical layer specific [1, 3, 29], the mPFC and somatosensory cortex were divided into their respective layers as previously described [21, 30–33]. In the mPFC, there were no differences in the number of Tbr1- or Satb2-positive excitatory neurons per cortical layer (Fig. 1I, J). However, in the somatosensory cortex, the increase in Tbr1-positive neurons was specific to layers IV and VI in 3-month-old *Ptprd+/-* or *Ptprd-/-* mice compared to *Ptprd+/+* mice (Fig. 1K). For Satb2-positive neurons, the increase was observed in all cortical layers in the somatosensory cortex of 3-month-old *Ptprd-/-* mice compared to *Ptprd+/+* mice, except for cortical layer V, where *Ptprd+/-* mice had more Satb2-positive neurons compared to *Ptprd+/+* mice (Fig. 1L). These results indicate that loss of *Ptprd* in adult mice results in an increase in excitatory cortical neurons in brain areas associated with cognition.

**Fig. 2:**
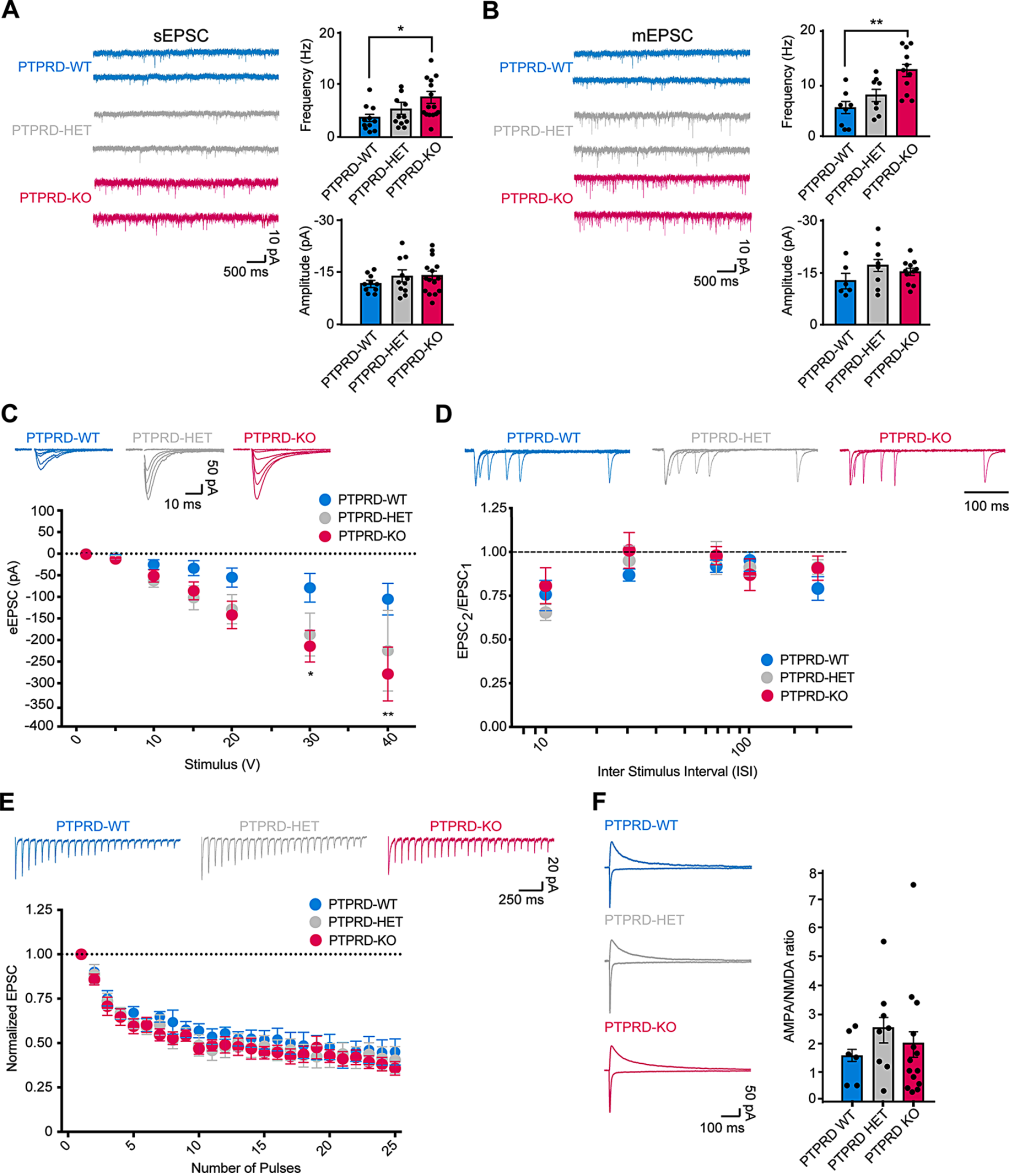
Excitatory synaptic transmission in layer 2/3 of the mPFC is disrupted in the *Ptprd-/- *mice. (**A**) Sample traces (left) and quantitative analysis (right) of sEPSC activity recorded from layer 2/3 pyramidal neurons in the mPFC in *Ptprd*+/+ (PTPRD-WT), *Ptprd+/-* (PTPRD-HET), and *Ptprd*-/- (PTPRD-KO) mice. A significant increase in the frequency but not in the amplitude of sEPSC in PTPRD-KO was detected. PTPRD-WT, *n* = 4 animals, 10 cells; PTPRD-HET, *n* = 5 animals, 11 cells; PTPRD-KO, *n* = 8 animals, 15 cells. **p < 0.01. (**B**) Miniature excitatory synaptic current (mEPSC) also shows an increase in the frequency but not in the amplitude in *Ptprd-/-* compared to *Ptprd+/+* and *Ptprd+/-* mice. PTPRD-WT, *n* = 5 animals, 8 cells; PTPRD-HET, *n* = 5 animals, 8 cells; PTPRD-KO, *n* = 6 animals, 11 cells. *p < 0.05. (**C**) Input/output function measured as the EPSP amplitude as a function of stimulus intensity also is increased in *Ptprd-/-* compared to *Ptprd+/+* and *Ptprd+/-* mice. PTPRD-WT, *n* = 3 animals, 6 cells; PTPRD-HET, *n* = 5 animals, 9 cells; PTPRD-KO, *n* = 5 animals, 7 cells. (**D**) Paired-pulse responses superimposed after subtraction of the first pulse at 10, 30, 70, 100, and 300 ms interstimulus intervals (ISI) are similar between genotyping. PTPRD-WT, *n* = 4 animals, 6 cells; PTPRD-HET, *n* = 6 animals, 8 cells; PTPRD-KO, *n* = 5 animals, 8 cells. (**E**) Short-term synaptic responses (top) and normalized summary data (bottom) evoked by a burst of 25 stimuli at 14 Hz are also similar between genotyping. PTPRD-WT, *n* = 3 animals, 6 cells; PTPRD-HET, *n* = 4 animals, 8 cells; PTPRD-KO, *n* = 4 animals, 9 cells. (**F**) AMPA/NMDA ratio shows no significant differences between genotyping. PTPRD-WT, *n* = 3 animals, 6 cells; PTPRD-HET, *n* = 3 animals, 8 cells; PTPRD-KO, *n* = 5 animals, 14 cells. In all graphs, the error bars denote SEMs

As an increased number of glutamatergic neurons is associated with altered synaptic function [33], we characterized the impact of the *Ptprd* ablation on the spontaneous synaptic activity in layer 2/3 pyramidal neurons in the mPFC. Firstly, we recorded spontaneous excitatory synaptic currents (sEPSCs) and found a significant increase in the frequency but not amplitude of sEPSCs in *Ptprd-/-* and not *Ptprd+/-* mice when compared to *Ptprd+/+* littermates (Fig. 2A). Similarly, an increase in the frequency of miniature excitatory synaptic currents (mEPSCs) but not in the amplitude was observed in *Ptprd+/-* or *Ptprd-/-* mice compared to *Ptprd+/+* mice (Fig. 2B). Secondly, we analyzed the evoked synaptic transmission mediated by AMPA receptors by generating input–output curves. We observed an increased response at high voltage inputs in *Ptprd-/-* mice compared to *Ptprd+/+* (Fig. 2C), suggesting that excitatory transmission is augmented in the *Ptprd-/-* mice. Thirdly, we examined whether the effect of the PTPRD ablation alters synaptic release probability by estimating two principal forms of short-term synaptic plasticity: paired-pulse ratio (PPR) and use-dependent depression. However, both PPR (Fig. 2D) and synaptic depression in response to a stimulus train of 25 stimuli at 14 Hz (Fig. 2E) remained unchanged between genotypes. Finally, we measured the AMPA/NMDA ratio and found no significant differences between genotypes (Fig. 2F). Together, these results suggest that the increase in excitatory transmission cannot be accounted for by changes in postsynaptic receptor numbers or changes in release probability, but rather, could be due to an increase in the number of synaptic contacts in *Ptprd-/-* mice.

### *Ptprd*+/- or *Ptprd*-/- mice display an increase in GABAergic neurons and aberrant inhibitory synaptic transmission in the adult cortex

**Fig. 3:**
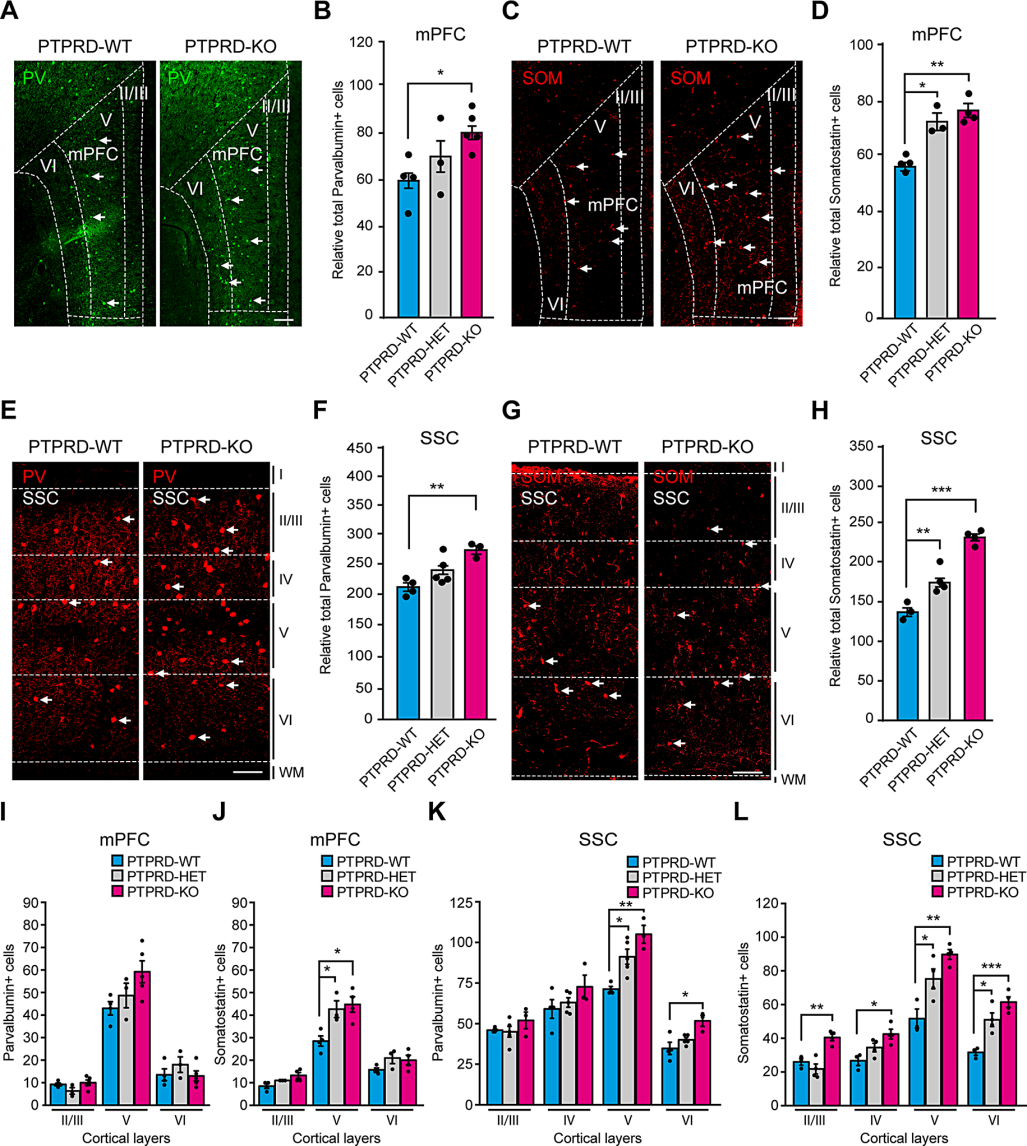
*Ptprd+/- or Ptprd-/-* mice show an increase of GABAergic neurons in the mPFC and somatosensory cortex at 3 months. (**A-H**) Coronal sections of the mPFC or somatosensory cortex of *Ptprd*+/+ (PTPRD-WT) and *Ptprd*-/- (PTPRD-KO) 3-month-old animals were immunostained against parvalbumin (**A, E**) or somatostatin (**C, G**). Relative total parvalbumin-positive cells in the mPFC **(**for **B**, PTPRD-WT, *n* = 4; PTPRD-HET, *n* = 3; PTPRD-KO, *n* = 5**)** or somatosensory cortex **(**for **F**, PTPRD-WT, *n* = 4; PTPRD-HET, *n* = 5; PTPRD-KO, *n* = 3**)** of *Ptprd*+/+, *Ptprd*+/- (PTPRD-HET), and *Ptprd*-/- 3-month-old mice. Relative total of somatostatin-positive cells (arrows head) in the mPFC **(**for **D**, PTPRD-WT, *n* = 4; PTPRD-HET, *n* = 3; PTPRD-KO, *n* = 4**)** or somatosensory cortex **(**for **H**, PTPRD-WT, *n* = 3; PTPRD-HET, *n* = 4; PTPRD-KO, *n* = 4**)** of *Ptprd*+/+, *Ptprd*+/- and *Ptprd*-/- 3-month-old mice. In (**A**) and (**C**), the dotted line shows mPFC and specific cortical layers. **(I, J)** Parvalbumin (**I**) or Somatostatin (**J**) positive neurons were quantified per cortical layers II/III, V and VI in the mPFC. **(K, L)** Parvalbumin (**K**) or Somatostatin (**L**) positive neurons were quantified per cortical layers II/III, IV, V and VI in the somatosensory cortex. mPFC = medial prefrontal cortex. SSC = somatosensory cortex. PV = parvalbumin. SOM = somatostatin. WM = white matter. In all the images, scale bars represent 200 μm, and arrows show positive cells. *p < 0.05; **p < 0.01; ***p < 0.001. In all graphs, the error bars denote SEMs

*Ptprd* is expressed in subcortical structures during brain development [34] and is important for GABAergic synaptic differentiation [35–37]. However, its role in regulating the generation of GABAergic neurons has not been described. We therefore evaluated whether loss of *Ptprd* expression could also alter the number of GABAergic neurons in the adult mouse cortex. To address this, we performed immunostaining for parvalbumin (Fig. 3A) and somatostatin (Fig. 3C), two major types of cortical GABAergic neurons [2], at the mPFC. We found that *Ptprd-/-* mice had more parvalbumin-positive (Fig. 3B) and somatostatin-positive neurons (Fig. 3D) than wild-type littermates in the mPFC. Additionally, we immunostained for parvalbumin (Fig. 3E) and somatostatin (Fig. 3G) at the somatosensory cortex. Quantification showed that *Ptprd-/-* mice had more parvalbumin- (Fig. 3F) and somatostatin-positive neurons (Fig. 3H). In addition, *Ptprd* heterozygous mice had more somatostatin-positive neurons (Fig. 3H), indicating that GABAergic neurons are increased in the cortex of 3-month-old *Ptprd-/-* mice.

We then evaluated the number of inhibitory neurons per layer. We found no differences in the number of parvalbumin-positive neurons in the mPFC per layer across genotypes (Fig. 3I). However, when we analyzed the somatostatin-positive neurons, we found that they were increased specifically in the mPFC layer V of 3-month-old *Ptprd+/-* or *Ptprd-/-* compared to *Ptprd+/+* mice (Fig. 3J). Next, we performed a similar analysis in the somatosensory cortex and found that parvalbumin-positive neurons were specifically increased in layers V and VI (Fig. 3K). For somatostatin-positive neurons, the increase was observed in all cortical layers in the somatosensory cortex of 3-month-old *Ptprd-/-* compared to *Ptprd+/+* mice, except for cortical layers V and VI, where *Ptprd+/-* mice also had more somatostatin-positive neurons compared to *Ptprd+/+* mice (Fig. 3L).

**Fig. 4:**
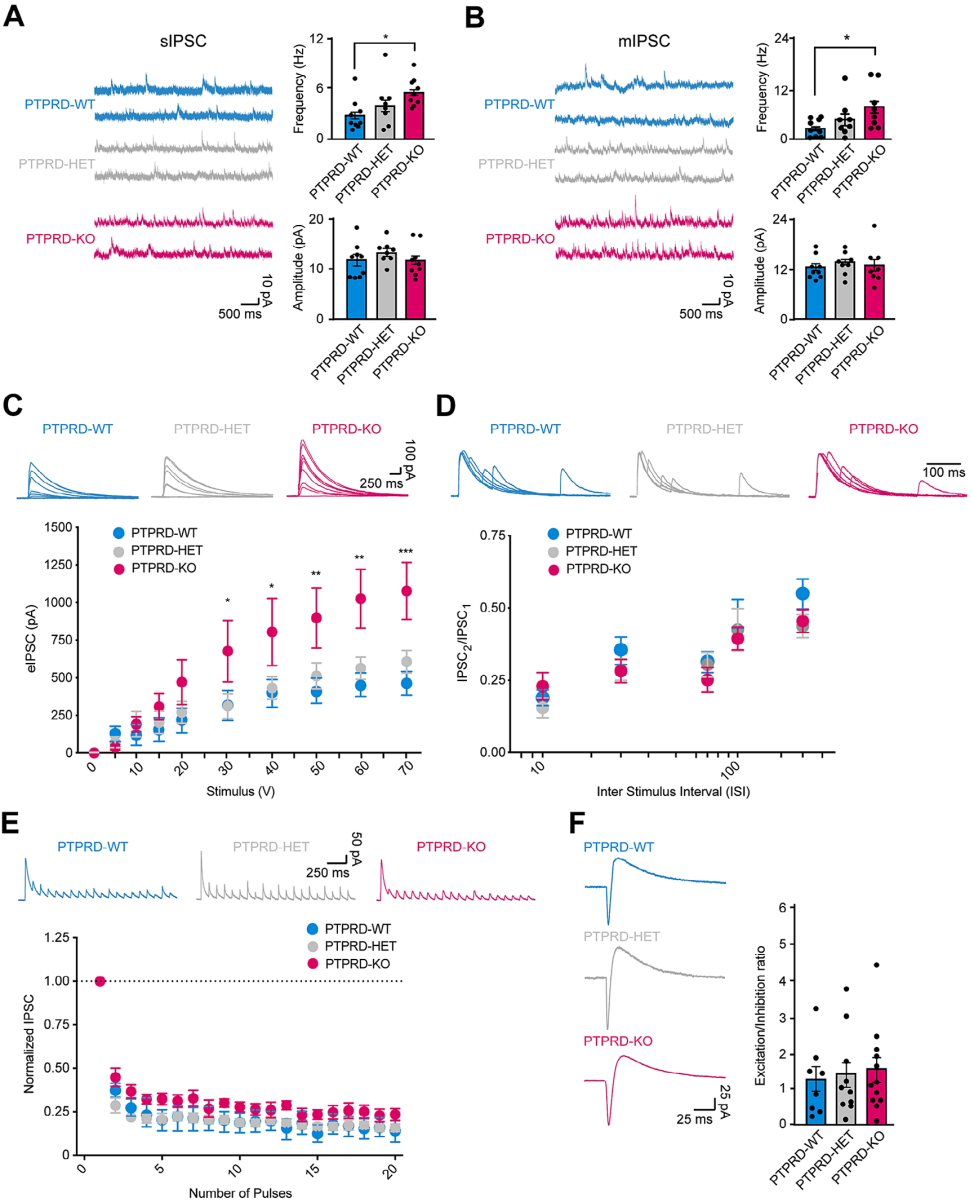
Inhibitory synaptic transmission in layer 2/3 of the mPFC is impaired in the *Ptprd-/- *mice. (**A**) Representative traces (left) and quantitative analysis (right) of sIPSC activity recorded from layer 2/3 pyramidal neurons in the mPFC in *Ptprd*+/+ (PTPRD-WT), *Ptprd+/-* (PTPRD-HET), and *Ptprd*-/- (PTPRD-KO). PTPRD-WT, *n* = 5 animals, 9 cells; PTPRD-HET, *n* = 6 animals, 8 cells; PTPRD-KO, *n* = 4 animals, 9 cells. *p < 0.05. (**B**) Representative traces (left) and quantitative analysis (right) of mIPSC activity recorded from layer 2/3 pyramidal neurons in the mPFC in *Ptprd*+/+ (PTPRD-WT), *Ptprd+/-* (PTPRD-HET), and *Ptprd*-/- (PTPRD-KO). PTPRD-WT, *n* = 4 animals, 9 cells; PTPRD-HET, *n* = 4 animals, 8 cells; PTPRD-KO, *n* = 4 animals, 8 cells. *p < 0.05. (**C**) Evoked IPSC amplitudes as a function of stimulus intensity plotted as input/output curves show an increase in *Ptprd-/-* compared to *Ptprd+/+* and *Ptprd+/-* mice. Significant changes between *Ptprd+/+* and *Ptprd-/-* groups are as “*”. *p < 0.05, **p < 0.01, and ***p < 0.001. PTPRD-WT, *n* = 4 animals, 7 cells; PTPRD-HET, *n* = 5 animals, 9 cells; PTPRD-KO, *n* = 4 animals, 8 cells. (**D**) Paired-pulse responses at 10, 30, 70, 100, and 300 ms interstimulus intervals (ISI) are similar between genotyping. PTPRD-WT, *n* = 4 animals, 6 cells; PTPRD-HET, *n* = 4 animals, 6 cells; PTPRD-KO, *n* = 5 animals, 8 cells. (**E**) Short-term synaptic responses (top) and normalized summary data (bottom) evoked by a burst of 20 stimuli at 10 Hz are also similar between genotyping. PTPRD-WT, *n* = 4 animals, 7 cells; PTPRD-HET, *n* = 6 animals, 10 cells; PTPRD-KO, *n* = 4 animals, 7 cells. (**F**) Excitatory and inhibitory balances show no significant differences between genotyping. PTPRD-WT, *n* = 4 animals, 8 cells; PTPRD-HET, *n* = 4 animals, 10 cells; PTPRD-KO, *n* = 5 animals, 12 cells. In all graphs, the error bars denote SEMs

As the increase in glutamatergic neurons was coupled with increase synaptic activity, we evaluated whether the increase in GABAergic neurons observed in *Ptprd-/-* mice was associated with an increase in inhibitory synaptic transmission by recording spontaneous inhibitory synaptic currents. As we found with excitatory synaptic transmission (Fig. 2), we observed an increase in the frequency but not amplitude of spontaneous inhibitory synaptic currents (sIPSCs) in *Ptprd-/-* but not *Ptprd+/-* mice when compared to *Ptprd+/+* littermates (Fig. 4A). Similarly, there was an increase in the frequency but not amplitude of miniature synaptic currents (mIPSCs) in *Ptprd-/-* mice (Fig. 4B). Next, we evaluated evoked inhibitory input-output curves and found an enhancement in evoked IPSCs in *Ptprd-/-* compared to *Ptprd+/+* mice (Fig. 4C), suggesting an increase in GABAergic synaptic transmission. To further evaluate the effect of the PTPRD ablation on GABAergic synaptic function, we measured PPR and use-dependent depression and found that both PPR and synaptic depression in response to a stimulus train of 20 stimuli at 10 Hz remained unchanged between genotypes (Fig. 4D, E), indicating that the increase in GABAergic function is independent of changes in release probability.

Finally, we tested whether the enhanced excitatory and inhibitory inputs onto layer 2/3 pyramidal neurons in *Ptprd-/-* mice altered the excitatory/inhibitory balance of these neurons. We recorded simultaneously evoked EPSC and IPSCs by holding pyramidal neurons at − 35 mV and found no significant differences in the excitation/inhibitory ratio among genotypes (Fig. 4F), indicating that the increase in synaptic function observed in *Ptprd-/-* mice does not alter the excitatory/inhibitory balance in the mPFC.

### *Ptprd*+/- or *Ptprd*-/- mice do not show impairments in learning and memory or increased anxiety

**Fig. 5:**
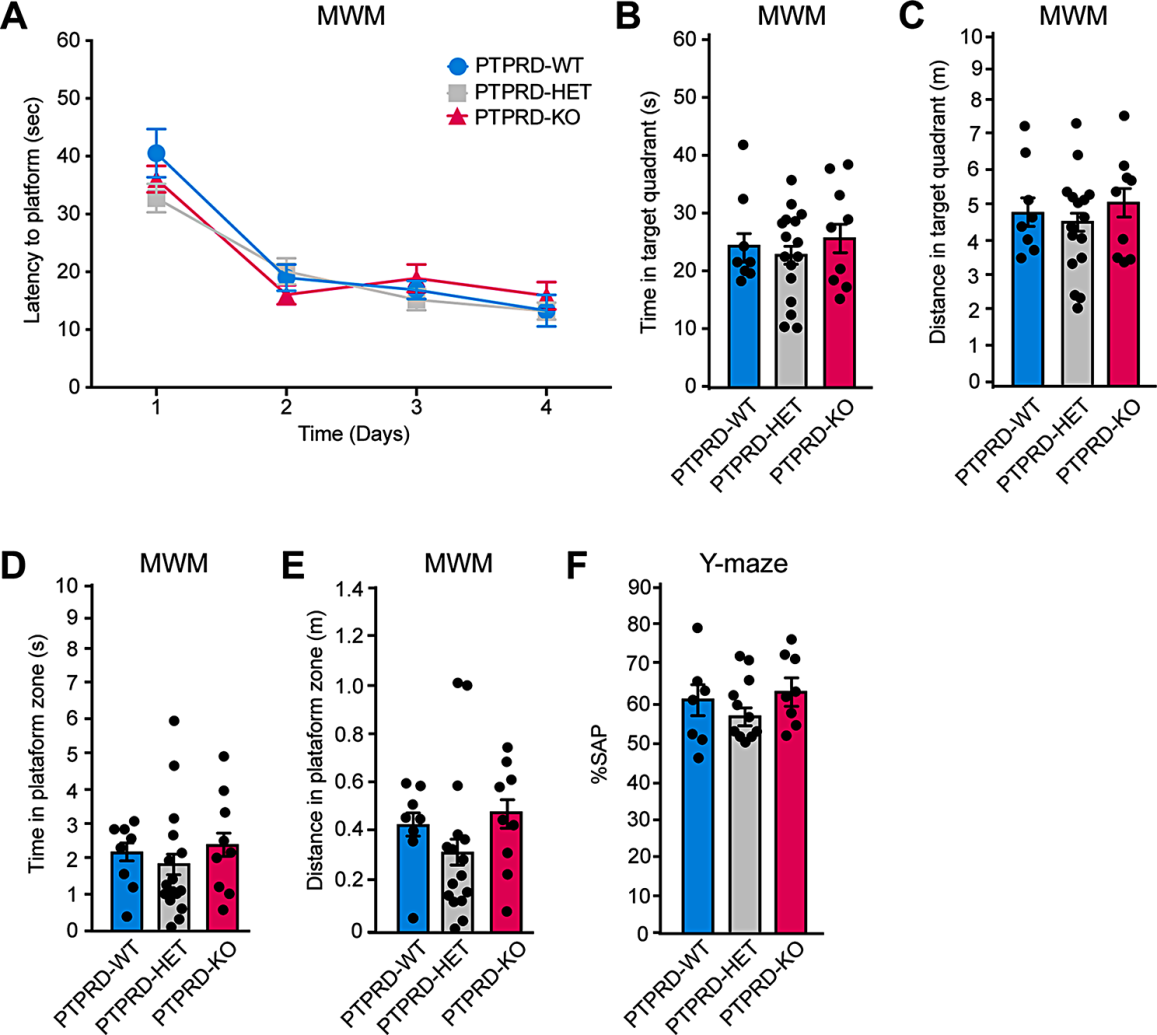
Heterozygous or homozygous *Ptprd *mice showed no learning and memory deficits. (**A-E**) 3-month-old *Ptprd+/+* (PTPRD-WT), *Ptprd*+/- (PTPRD-HET), and *Ptprd-/-* (PTPRD-KO) mice were tested in the Morris water maze (MWM) test. **(A)** Latency escape during the 4 training days. **(B)** Time spent by the animals swimming in the target quadrant area on test day (day 5). **(C)** Distance traveled in the target quadrant zone on test day. **(D)** Time spent by the animals swimming in the platform area on test day. **(E)** Distance traveled in the platform zone on test day. PTPRD-WT, *n* = 8; PTPRD-HET, *n* = 16; PTPRD-KO, *n* = 9. **(F)** 3-month-old *Ptprd+/+*, *Ptprd+/-*, and *Ptprd-/-* mice were tested in the Y-maze test. Percentage of preference for spontaneous alternations (SAP). PTPRD-WT, *n* = 6; PTPRD-HET, *n* = 11; PTPRD-KO, *n* = 8. In all graphs, the error bars denote SEMs

Mutation or aberrant expression of genes associated with neuroanatomical alterations are thought to have profound consequences in adulthood as they affect the connectivity between neurons, promoting brain disorders [5]. As *PTPRD* mutations have been associated with brain disorders that include cognitive impairments, we evaluated whether *Ptprd+/-* or *Ptprd-/-* mice have learning and memory deficits by performing the Morris Water Maze test. We found that *Ptprd-/-* or *Ptprd+/-* exhibited normal latency escape (Fig. 5A). Also, they showed normal time spent (Fig. 5B) and distance (Fig. 5C) in the target quadrant as well as in the time spent (Fig. 5D) and distance (Fig. 5E) in the area where the platform was located compared to *Ptprd+/+ mice*. To confirm these observations, we performed the Y-maze test, and found that *Ptprd+/-* or *Ptprd-/-* mice showed no difference in the percentage of spontaneous alternation preference as compared to *Ptprd*+/+ animals (Fig. 5F). These results indicate that *Ptprd+/-* or *Ptprd-/-* mice do not present with spatial learning and memory deficits.

**Fig. 6:**
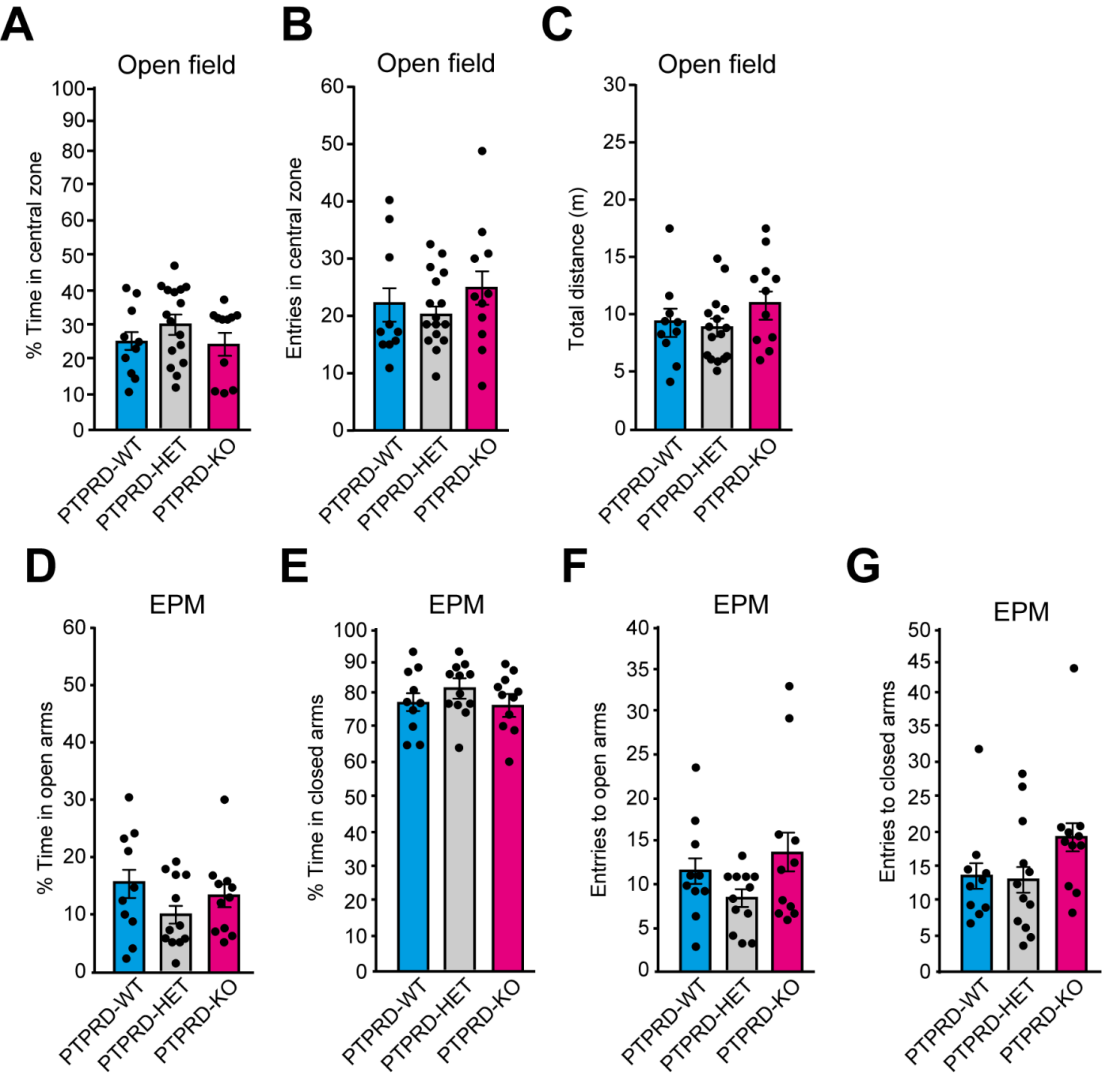
Loss of one or both *Ptprd *alleles does not induce anxious behavior. (**A-C**) 3-month-old *Ptprd+/+* (PTPRD-WT), *Ptprd+/-* (PTPRD-HET), and *Ptprd-/-* (PTPRD-KO) mice were tested in open-field anxiety test. **(A)** Percentage of the total time that the mice spent in the central zone. (**B**) Number of times that the animals enter the central zone. (**C**) Total distance traveled. PTPRD-WT, *n* = 10; PTPRD-HET, *n* = 16; PTPRD-KO, *n* = 11. (**D-G**) 3-month-old *Ptprd*+/+, *Ptprd*+/-, and *Ptprd*-/- mice were tested in the elevated plus maze test (EPM). (**D**) Percentage of the total time spent in open arms. (**E**) Percentage of the total time spent in closed arms. **(F)** Number of times that the animals enter the open arms. (**G**) Number of times that the animals enter the closed arms. PTPRD-WT, *n* = 10; PTPRD-HET, *n* = 12; PTPRD-KO, *n* = 11. In all graphs, the error bars denote SEMs

As individuals with *PTPRD* variants exhibit anxiety [38] we evaluated if *Ptprd+/-* or *Ptprd-/-* mice showed increased anxiety using the open-field and elevated plus maze tests [26, 39]. In the open field test, we observed no differences between *Ptprd+/-* or *Ptprd-/-* and *Ptprd*+/+ animals in the time spent in the central zone (Fig. 6A) and the number of times that mice entered this area (Fig. 6B). In addition, none of the animal groups presented differences in the distance covered, indicating that the mice did not have motor deficits (Fig. 6C). In the elevated plus maze test, there were no differences between genotypes in terms of the percentage of time spent in the open (Fig. 6D) and closed arms (Fig. 6E) or in the number of entries to these arms (Fig. 6F, G). Together, these results suggest that losing one or both alleles of *Ptprd* does not result in learning and memory impairment or aberrant anxious behavior.

### *Ptprd*+/- or *Ptprd*-/- mice exhibit autistic-like behaviors

**Fig. 7:**
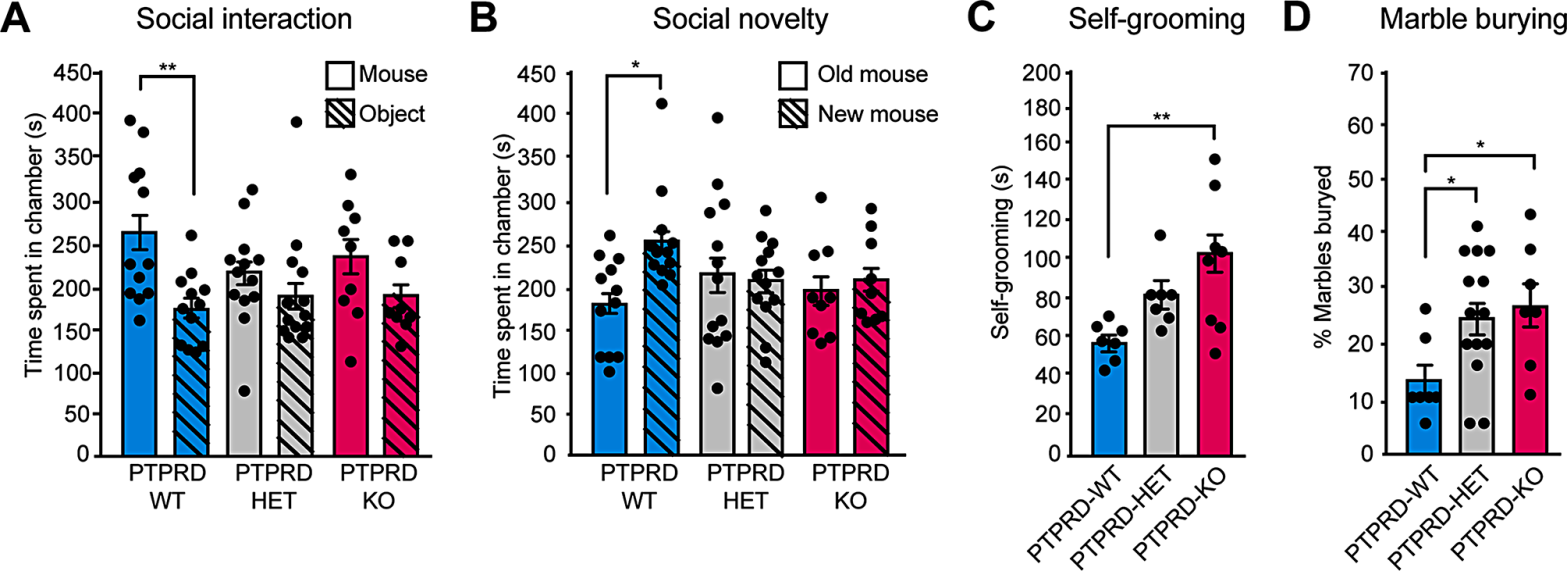
*Ptprd+/-* or *Ptprd-/-*mice showed autistic-like behaviors. (**A-D**) 3-month-old *Ptprd*+/+ (PTPRD-WT), *Ptprd*+/- (PTPRD-HET), and *Ptprd*-/- (PTPRD-KO) mice were tested for sociability (**A**) and social novelty (**B**), self-grooming (**C**) or marble burying (**D**). (**A**) Social Interaction Test was measured in the three-chamber test. The graph shows the cumulative duration of *Ptprd*+/+, *Ptprd*+/-, and *Ptprd*-/- mice in the mouse or object chambers. PTPRD-WT, *n* = 12; PTPRD-HET, *n* = 13; PTPRD-KO, *n* = 9. (**B**) Social Novelty Test was assessed in the three-chamber test. The graph shows the cumulative duration of *Ptprd*+/+, *Ptprd*+/-, and *Ptprd*-/- mice in the old mouse and new mouse chambers. PTPRD-WT, *n* = 12; PTPRD-HET, *n* = 13; PTPRD-KO, *n* = 9. *p < 0.05; **p < 0.01. **(C, D)** Repetitive behavior was evaluated by the time spent in spontaneous self-grooming **(C)** or marble-burying behaviors (**D**). (**C**) Cumulative time in which *Ptprd*+/+, *Ptprd*+/-, and *Ptprd*-/- mice did self-grooming behavior in a total of 10 min. PTPRD-WT, *n* = 7; PTPRD-HET, *n* = 7; PTPRD-KO, *n* = 8. (**D**) Percentage of marbles buried by *Ptprd*+/+, *Ptprd*+/-, and *Ptprd*-/- mice after 30 min. PTPRD-WT, *n* = 7; PTPRD-HET, *n* = 14; PTPRD-KO, *n* = 7. *p < 0.05; **p < 0.01. In all graphs, the error bars denote SEMs

*PTPRD* variants have also been associated with ASD [40–43]. Therefore we asked whether the loss of one or both *Ptprd-/-* alleles induces autistic-like behaviors. To address this, we evaluated social behavior using the three-chamber test for social interaction and novelty [44]. In this test, wild-type rodents prefer to spend more time with another rodent (sociability) and will investigate a novel intruder more than a familiar one (social novelty). To evaluate the sociability of *Ptprd-/-* mice, they were placed in a context where they could interact with a caged mouse or an inanimate object, each in a different chamber. We observed that *Ptprd-/-* or *Ptprd+/-* mice, unlike *Ptprd*+/+ animals, did not show a preference to spend more time exploring the chamber containing the caged mouse, indicating a social deficit (Fig. 7A). The inanimate object was then replaced by another mouse (new mouse) and the caged mouse was kept in the same chamber (old mouse). *Ptprd-/-* or *Ptprd+/-* mice did not discriminate between the old and new mouse, indicating a deficit in social novelty compared to *Ptprd+/+* mice (Fig. 7B).

Next, we evaluated repetitive and stereotyped behaviors. Self-grooming behavior is often altered in mouse models of ASD [45]. Thus, we recorded mice from below through a transparent box to allow the visualization and further analysis of self-grooming. *Ptprd*-/- mice increased their time self-grooming as compared to *Ptprd+/+* mice (Fig. 7C). Finally, we used the marble burying test, a test widely used to assess repetitive behavior in mouse models of compulsive disorder and ASD [46, 47]. The test assesses the percentage of marbles that mice bury in 30 min, which indirectly reflects the degree of repetitive digging behavior. We found that *Ptprd-/-* or *Ptprd+/-* mice showed a significant increase in buried marbles as compared to *Ptprd+/+* mice (Fig. 7D). Therefore, our behavioral results suggest that loss of one or both *Ptprd* alleles induces autistic-like behaviors.

## Discussion

Here, we analyzed how loss of *Ptprd* expression impacts the generation of glutamatergic and GABAergic neurons and synaptic transmission in the cerebral cortex, potentially contributing to the emergence of behavioral impairments in adulthood. Mutations or aberrant expression of this gene have previously been associated with various neurodevelopmental disorders such as obsessive-compulsive disorder, schizophrenia, and ASD [14]. In this regard, we reported that *Ptprd* is a key regulator of mouse embryonic neurogenesis, controlling the numbers of neurogenic intermediate progenitor cells and, ultimately, the number of neonatal cortical neurons [11, 12]. Here, we found that the developmental increase of excitatory neurons persisted through adulthood, affecting excitatory synaptic function in the mPFC. Moreover, loss of *Ptprd* expression resulted in an increase in inhibitory GABAergic neurons and inhibitory synaptic function. Finally, adult *Ptprd-/-* mice displayed autistic-like behaviors, suggesting that ablation of *Ptprd* not only influences the number and function of cortical neurons but also behavior.

What accounts for the increase in glutamatergic neurons in adult *Ptprd-/-* mice? We propose that it is directly related to the increase in Tbr2-positive intermediate precursor cells in these mice during embryogenesis that we previously reported [11, 12]. There is abundant evidence that ASD-associated genes exhibit cell cycle changes in neural precursor cells during brain development, culminating in an alteration in the total number of glutamatergic neurons [48]. For example, Fang et al. [33], reported that the increase in Tbr2-positive intermediate precursors that in turn produces increased amounts of glutamatergic neurons, was sufficient to elicit alterations in synaptic function, interneuron mislocalization, and the appearance of ASD-like behaviors. The genes affected in animal models of ASD in which the number of Tbr2-positive cells is increased are many [48]. Therefore, given that the pathophysiological mechanisms that trigger the onset of ASD-like behaviors include alterations in the production of glutamatergic neurons [33, 48] like those observed in the mPFC and somatosensory cortex of *Ptprd+/-* or *Ptprd-/-* mice, this could, at least in part, explain the ASD-like behavior in these animals.

Ptprd participates both as an adhesion molecule promoting synaptic formation and differentiation, and a phosphatase regulating the activity of receptors involved in synaptic transmission such as TrkB [14]. The role of Ptprd in synaptic function in cultured hippocampal neurons is conflicting, as the absence of *Ptprd* has been reported to either not alter synaptic formation or transmission [18, 19] or decrease excitatory synaptic transmission [17]. Our results obtained in acute mPFC slices from adult *Ptprd*-/- mice indicate an increase in both excitatory and inhibitory synaptic activity. While this increase was not due to changes in the number of postsynaptic receptors or release probability, it is possible that these differences could be due to increased number of synaptic contacts in the *Ptprd-/-* mPFC.

*Ptprd+/-* or *Ptprd-/-* mice showed autistic-like behaviors. ASD is characterized by social deficits and repetitive behavior [49]. Consistent with this idea, *Ptprd+/-* or *Ptprd-/-* mice present deficits in sociability with a conspecific and the preference for interaction with a new individual, supporting findings of mutations in *PTPRD* linked to ASD risk [40–43]. Moreover, *Ptprd-/-* mice have increased self-grooming and marble-burying behavior, core symptoms of ASD. However, our findings do differ from those reported by Ho et al. [50] that did not report changes in the time invested in self-grooming. These differences likely reflect the different experimental paradigms used. We evaluated instinctively self-grooming behavior without stimuli to promote it. Ho et al. [50] quantified self-grooming time after spraying the animals with a sucrose solution to encourage the indicated behavior. We propose that artificially stimulating self-grooming could mask normal behavior in *Ptprd+/+* animals. We also evaluated the number of the marbles buried that represents the occurrence of repetitive behavior, while Ho et al. [50] evaluated the time and number of times the experimental animals spent performing the digging process. While the experimental paradigms used by both groups are valid, our findings of increased repetitive behavior in the *Ptprd-/-* mice do indeed indicate that they exhibit ASD-like behavior.

Uetani et al. [51] and Drgonova et al. [52] reported impairments of spatial learning and memory in *Ptprd-/-* mice which we did not observe. While the Uetani et al. [51], Drgonova et al. [52], and our mice were in the same C57BL/6J genetic background, there were evident differences in the mouse models. The mice of those groups showed growth retardation and weight loss attributed to problems in food intake. While their *Ptprd-/-* mice lived for approximately one year, our animals aged normally. To confirm the lack of spatial learning and memory impairments, we also evaluated our animals in the Y-maze test, which showed no cognitive deficits associated with learning and memory. Our findings regarding the lack of anxious behavior in *Ptprd-/-* mice were similar to the results reported by Park et al. [17] and Ho et al. [50], who used the same *Ptprd*-/- model as that by Uetani et al. [51] and Drgonova et al. [52]. The findings will need to be resolved in future studies with neural precursor specific knockouts of *Ptprd*.

## Conclusions

Our data, together with previous findings [11, 12], show that several phenotypic features normally associated with ASD were present in mice lacking *Ptprd*, including deficits in neural precursor cell proliferation, increased neurogenesis, neuronal mislocalization [48, 53], alterations in the number and function of glutamatergic and GABAergic neurons [54], and social deficits and repetitive behavior [55]. These findings lead us to propose that the absence of *Ptprd* expression disrupts the generation and functioning of glutamatergic and GABAergic cortical neurons, inducing the appearance of ASD-like behavior in adulthood in mice. Our results suggest a possible explanation for why variants in *PTPRD* result in brain disorders.

## Data Availability

The datasets used and/or analyzed during the current study are available from the corresponding author on reasonable request.
